# Biodegradable Fiber Preparation Technique to Meet Industrial Requisites Through Sheath-Core Melt-Spinning

**DOI:** 10.3390/polym17040527

**Published:** 2025-02-18

**Authors:** Jin Yoo, Ga Hee Kim, Jun-Yeop Shim, Seok Eon Lee, Shi Hyeong Kim, Taehwan Lim, Jun Sik Son

**Affiliations:** 1 Division of Chemical Engineering and Bioengineering, Kangwon National University, Chuncheon 24341, Gangwon-do, Republic of Korea; dbwls01@kangwon.ac.kr; 2 Korea Textile Development Institute, Daegu 41842, Republic of Korea; kgh@textile.or.kr; 3 R&F Chemical Co., Ltd., Hanam 12925, Gyeonggi-do, Republic of Korea; shim@rnfchemical.com; 4 FITI Testing & Research Institute, Cheongju 28115, Chungcheongbuk-do, Republic of Korea; dltjrdjs@fitiglobal.com; 5 Department of Advanced Textile R&D, Korea Institute of Industrial Technology (KITECH), Ansan 15588, Gyeonggi-do, Republic of Korea

**Keywords:** biodegradable fibers, thermoplastic starch, biodegradability, spinnability, melt-spinning

## Abstract

Biodegradable polymers are essential for sustainable plastic life cycles and contribute to a carbon-neutral society. Here, we explore the development of biodegradable fibers with excellent mechanical properties using polypropylene (PP) and thermoplastic starch (TPS) blends. To address the inherent immiscibility between hydrophobic PP and hydrophilic TPS, hydrophilic modification and a masterbatch approach were employed. Melt-spinning trials demonstrated that the modified PP and TPS blends (mPP/TPS) exhibited excellent spinnability and processability comparable to virgin PP. A sheath-core configuration was introduced to enhance biodegradability while maintaining structural stability, with an mPP-rich part as the core and a TPS-rich part with a biodegradable promoter (BP) as the sheath. SEM and DSC analyses confirmed strong interfacial compatibility, uniform fiber morphology, and single melting points, indicating no phase separation. Mechanical testing showed that the sheath-core fibers met industrial requirements, achieving a tenacity of up to 2.47 gf/den and tensile strain above 73%. The addition of a BP increased the biodegradability rate, with PP/TPS/BP fibers achieving 65.93% biodegradation after 115 days, compared to 37.00% for BP-free fibers. These results demonstrate the feasibility of blending petroleum-based polymers with bio-based components to create fibers that balance biodegradability, spinnability, and mechanical performance, offering a sustainable solution for industrial applications.

## 1. Introduction

Biodegradable polymers promote sustainable plastic life cycles and contribute to achieving a carbon-neutral society, a key goal for modern society [1,2]. Many studies focus on developing biodegradable polymers that are easy to degrade, yet durable, mechanically strong, and non-toxic [3,4]. Among these, poly(lactic acid) (PLA) is the most spotlighted polymer material due to its bio-based origin and relatively high mechanical properties [5]. Thus, numerous studies have explored the practical use of PLA across various industrial fields [6,7,8,9]. However, it is already known that its strict biodegradation conditions, relatively short repeating unit, and the presence of a methyl side group cause brittleness in PLA and result in a slow crystallization rate [10,11,12].

To obtain an ideal sustainable and carbon-neutral material, it is essential to address multiple factors, including mechanical properties, processability, cost-effective benefit, less toxicity, as well as biodegradability. Therefore, extensive research is conducted to find better biodegradable materials or optimal biopolymer blends, such as [13,14,15,16]. Thermoplastic starch (TPS) is one of the attractive biodegradable materials due to its low cost, bio-based origin, and non-toxicity [17]. Thus, many studies have focused on developing improved starch-based materials or blending TPS with various additives to enhance mechanical properties and thermal processability [18,19]. These are critical challenges that must be addressed for practical and industrial applications.

For the commercial utilization of the polymers, the fiber spinning process, especially, by the melt-spinning process, is regarded as crucial as a film-based plastic application. Melt-spun fiber is an advantageous material with diverse applications, offering several unique benefits. First and foremost, it is already certified for industrial use, such as high customizability and cost-effective manufacturing due to its fast fabrication benefit. The fiber is therefore highly versatile, as they can be easily transformed into various forms through post-processing techniques, including knitting or weaving, to meet specific application needs [20,21]. Also, its mechanical properties can be adjusted during fabrication through post-drawing processes, allowing for designed performance [22,23]. Furthermore, its one-dimensional structure enables its use as a component in polymer composites, providing flexibility to achieve both anisotropic and isotropic properties, depending on design requirements [24,25,26]. Hence, extensive study has focused on the melt-spinning of biodegradable polymers to exploit their environmental benefits in fiber-based applications [27,28,29,30].

Despite the many studies, achieving commercial biodegradable fibers preparation by melt-spinning remains challenging. Most biodegradable polymers have obstacles to manage the thermally induced viscoelastic transitions of these materials, which are intrinsically linked to their chemical structure and monomer origins [27,31,32]. When processing temperatures are evaluated, the polymer chains undergo alterations in relaxation dynamics, crystallization behavior, and molecular entanglement density. These should affect the fine-tuning of melt-spinning parameters required for stable and continuous fiber fabrication. As a result, the consistency of biodegradable polymer-based fiber preparation is hindered, and we prefer other methods that are also considered to overcome the suggested hindrances.

The development of biodegradable fibers based on entirely opposite approaches is also being explored to address the previous challenge. For example, conventional petroleum-based aliphatic polymers, such as polypropylene (PP) and poly(ethylene terephthalate) (PET), already exhibit excellent processability and desirable mechanical performance that are key requirements for industrial use [33,34]. If these materials can be directly modified to produce biodegradable fibers, it might be possible to overcome the limitations related to current bio-based polymers.

Reflecting this trend, oxo-biodegradable polymers have recently gained attention as promising approaches to improve fiber processability while also enhancing the biodegradability of conventional polymers. These polymers are manufactured by incorporating an additive called a “biodegradation promoter” into the existing polymer matrix, where BP acts as a pro-oxidant within the polymer structure [35,36,37,38]. In oxo-biodegradable polymers, the BP generates free radicals under heat and light, triggering oxidative degradation and breaking polymer chains into smaller fragments. These fragments are more easily metabolized by microbes, accelerating their conversion into CO_2_, H_2_O, and biomass, thereby shortening degradation time [38,39]. Some studies have identified metal salts, such as iron, cobalt, and nickel, as effective catalysts for molecular-level degradation of plastics [40,41,42]. Also, a microbe-based biodegradable promoter has gained great attraction due to its ease of biodegradation mechanism under landfill anaerobic conditions as well [43,44,45,46]. The promoter helps initiate conditions favorable for certain microorganisms to metabolize by oxidation.

This approach offers a practical solution to promoting eco-sustainability and ultimately advancing toward a carbon-neutral society. However, potential toxicity and less bio-based origin contents must be addressed to ensure the sustainable application of biodegradable fibers [39,47,48]. Consequently, both suggested starch-based TPS and oxo-biodegradable polymer have their own and interestingly conflicting benefits; many studies have indicated that both require further modifications to become ideal biodegradable fibers suitable for industrial use.

In this study, we aimed to develop a biodegradable fiber material capable of meeting industrial requirements by combining the advantages of TPS and oxo-biodegradable conventional polymer. Our previous studies verified that melt-spinning is a highly precise and intricate process, presenting significant challenges when using unconventional polymers [49,50,51]. Additive-filled polymers often exhibit abnormal spinning yields; for example, high viscosity of TPS can hinder its consistent extrusion and its ability to blend effectively with other materials [18,19,52,53,54]. In addition, the biodegradation promoters incorporated into conventional polymers can act as impurities, disrupting the solid melt-spinning process. These additives block spinneret holes during processing, further complicating the fiber production.

**Figure 1:**
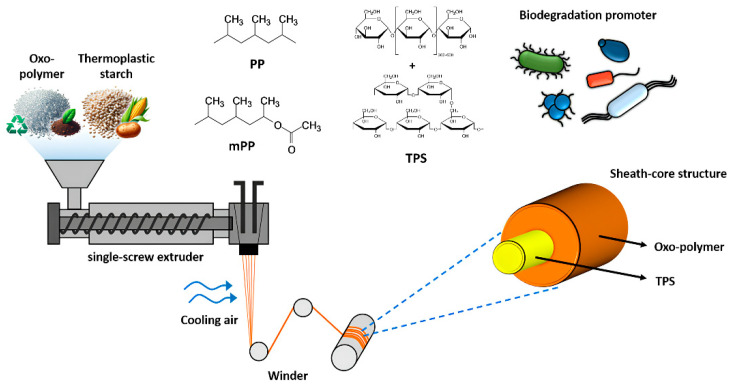
Schematic illustration for showing the concept of this study: oxo-polymer/TPS blend fibers with a sheath-core structure, exhibiting excellent spinnability and biodegradability, fabricated through the melt-spinning process.

Hence, we propose a strategy to fabricate biodegradable polymer-based fiber that shows both high processability and suitable mechanical performance. Many studies proved that increasing the proportion of TPS in the material enhances biodegradability, while incorporating high amounts of oxo-conventional polymer improves processability. To control the tradeoff, we designed a sheath-core fiber structure as a solution to maximize on the benefits of both components. The concept is illustrated in Figure 1.

Various studies already demonstrated that the concept of sheath-core fibers is effective for achieving multifunctionality at the same time. For example, Lim et al. developed poly(phenylene sulfide) (PPS) and poly(cyclohexanedimethanol terephthalate) (PCT) composite fibers using a sheath-core structure, combining the heat-resistant properties and economically affordable advantages, which are their own benefits, respectively [55]. Also, poly(styrene-butadiene-styrene) (SBS)/SBS-multiwall carbon nanotube (MWCNT) sheath-core fibers were developed, comprising the SBS as the core for elasticity and the SBS/MWCNT as the sheath for high mechanical and electrical performance [56]. Based on the examples, we applied the sheath-core design concept to our biodegradable polymer fibers. This approach enables us to harness the complementary properties of TPS and oxo-conventional polymers, achieving a balance between biodegradability and processability while ensuring mechanical strength for industrial applications.

Here, we conducted trials to develop fibers with improved elongation and mechanical performance after post-treatment. Using various parameters, such as melt flow index (MFI) and viscosity, as well as thermal properties, elongation behavior during the fabrication process, and the following mechanical performance of TPS/oxo-polymer fibers, we identified an optimal composition for producing melt-spinnable biodegradable fibers. Morphological analyses, including cross-sectional imaging, were performed to examine the fiber structure and validate the sheath-core design. Lastly, the biodegradability of the fibers was evaluated under simulated universal landfill conditions, demonstrating that the proposed sheath-core fibers effectively degrade in these environments.

## 2. Experimental Section

### 2.1. Materials

Two types of polypropylene (PP) were used in this study. Virgin PP (H7700, MFI = 34) was purchased from LG chemical (Seoul, Republic of Korea). For the hydrophilic-modified PP (mPP) and mPP/TPS masterbatch preparation, maleic anhydride (MA, 99%) and dicumyl peroxide (DCP, 98%) were purchased from Sigma-Aldrich and used without additional purification.

### 2.2. Modified Polypropylene Preparation

To enhance the compatibility among PP, TPS, and the biodegradation promoter (BP), PP was hydrophilically modified. Grafting MA onto PP using organic peroxide as a radical initiator regards as an effective approach to imparting polar functional groups onto the PP polymer chain, thereby improving its compatibility with polar polymers [57,58,59]. Hydrophilic modification of PP was carried out through an in situ reactive extrusion using a twin-screw extruder (BA-19, Bautek, Pocheon-si, Republic of Korea). The major temperature of the extruder was maintained at 190 °C, and the screw speed was set to 250 rpm. As the PP transported through the twin-screw extruder, it reacted with DCP and MA. During this process, DCP decomposed thermally at the set temperature to generate free radicals, which first bonded to PP polymer chains then reacted with MA, ultimately forming mPP [60].

### 2.3. Fiber Preparation

Three different types of blends (PP/TPS/BP, mPP/TPS/BP, mPP/TPS) and the sheath-core fibers were prepared using a melt-spinning process with a single-screw extruder (FET-MS-LAB-3, Fibre Extrusion Technology, UK). Virgin PP, mPP, TPS, and BP were used as raw materials, and their specific ratios are noted in Table 1. First, to improve the compatibility between PP and TPS, an mPP/TPS masterbatch was prepared in a twin-screw extruder (BA-19, Bautek, Republic of Korea) at 190 °C and 250 rpm. Primary trials to fabricate PP/TPS fibers and mPP/TPS/BP fibers were carried out using a single-screw extruder set to 200 °C and 7–9 rpm. All zones of the extruder were adjusted to the designated temperatures to optimize fiber formation and minimize thermal degradation. Fibers exhibited reduced mechanical strength due to thermal degradation at temperatures above the set extrusion temperature, while poor flowability at lower temperatures hindered proper fiber formation. Here, all the materials are fed up in the same time and feeder. The molten mixture was extruded through a spinneret with 36 multi-holes (length: 0.6 mm, diameter: 0.2 mm) to form filament fibers. The extruded fibers were air-cooled at room temperature before being collected by a winder set to a drawing ratio of up to 2.35 (always over 2.00). Sheath-core fibers were fabricated using two same melt-spinning setups with different spinnerets for the core and sheath. The equipment design is now disclosed due to confidentiality and secure reasons. Also, the design is not necessary in this study because we focused on the suggestion that higher biodegradable contents enhance the biodegradability.

### 2.4. Characterization of Morphologies

The lateral and cross-sectional morphologies of the fabricated fibers were examined using a filed-emission scanning electron microscope (FE-SEM, S-4300, Hitachi, Tokyo, Japan). Images were captured in secondary electron (SE) mode at an accelerating voltage of 7.0 kV. The cross-sections were prepared through microtomed.

### 2.5. Characterization of Rheological Properties

The viscosity and shear characteristics of the PP/TPS blends were analyzed using a capillary rheometer (Rheograph 25, GÖTTFERT Werkstoff-Prüfmaschinen GmbH, Buchen, Germany) at 190 °C, with a temperature tolerance of ±0.5 °C and a diameter of 15 mm.

### 2.6. Characterization of Thermal Properties

The thermal behavior and compatibility of the fibers were evaluated using differential scanning calorimetry (DSC, N-650, SCINCO, Seoul, Republic of Korea) ruled by the KS M ISO standard [61]. The temperature range was set from 22 to 300 °C. Melt flow characteristics were evaluated using a melt flow index (MFI) tester (MP600, Tinius Olsen, Salfords, UK).

### 2.7. Characterization of Mechanical Properties

The tenacity and tensile strain of the fibers were measured using a universal testing machine (Instron 3369, Norwood, MA, USA) ruled by the KS standard [62]. The specimens were 250 mm in length and 1 mm in width, with a total of 10 specimens (*n* = 10) tested to ensure statistical reliability. The test was conducted under the conditions of a speed set to 250 mm/min, a temperature of 22 ± 2–3 °C, and a humidity of 30 ± 2–3%. Other mechanical properties are tabulated in Table 1. “Denier” represents the fineness or density of the fiber, whereas tenacity and tensile strain indicate the tensile strength and elongation of the fibers, respectively. The MFI (measured at 230 °C/2.16 kg) shows the melt flow index of each blend, and the melting point reflects the flow characteristics of the polymers. For the PP8/TPS2/BP blend, stable fiber spinning was not achievable, so its MFI is solely reported; its quite high MFI value may limit spinnability. The modification of PP and the preparation of the masterbatch (M/B) contribute to enhancing the mechanical properties of the fibers by improving the compatibility between PP and TPS. This improvement is broadly reflected in Table 1, while more detailed analysis is provided in the results and conclusion sections.

### 2.8. Characterization of Biodegradability

The biodegradability of the sheath-core fibers was evaluated by measuring the cumulative CO_2_ emission, ruled by the ISO standard [63]. Compost served as the inoculum to mimic landfill condition, the reactor volume was 2.7 L, and the temperature was maintained at 58 °C. Cellulose was used as a positive reference material, and the test was carried out over 115 days. Lastly, biodegradability (%) was calculated from the produced CO_2_ amount and from that was subtracted the respiration CO_2_ amount determined from a test bed and the theoretically produced CO_2_ amount of that added to each sample.

## 3. Results and Discussions

### 3.1. Spinnability Confirmation

To determine the optimal melt-spinning conditions for PP/TPS composites and assess the impact after BP addition, five different fiber samples were firstly prepared using different melt-spinning techniques. The primary goal was to achieve both biodegradability and excellent mechanical properties suitable for industrial fibers. The properties of the fibers, including denier, tenacity, tensile strain, MFI, and melting point, were analyzed and are tabulated in Table 1. Each fiber sample was abbreviated based on the PP-to-TPS ratio in the blend and the presence of BP.

PP9/TPS1/BP and PP8/TPS2/BP composite fibers were initially fabricated via in situ extruder mixing to produce biodegradable fibers. TPS was blended with PP in a single-screw extruder during fiber preparation. However, both samples failed to achieve stable fiber spinning conditions due to poor compatibility between PP and TPS, resulting in non-homogeneous fiber formation. This incompatibility arises from the inherent hydrophobicity of PP hydrocarbon chains and the hydrophilicity of TPS hydroxyl groups, causing immiscible phases during extrusion [64,65,66].

**Figure 2:**
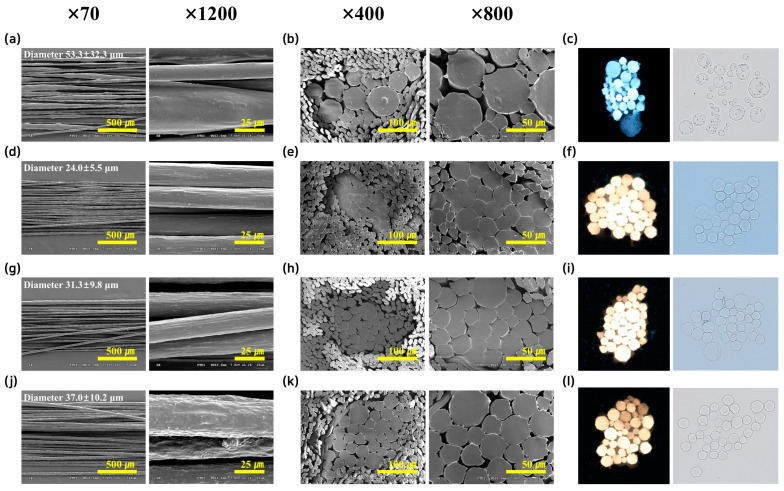
SEM images (**a**,**b**,**d**,**e**,**g**,**h**,**j**,**k**) and optical microscope images (**c**,**f**,**i**,**l**) of the fibers. Side view images of (**a**) PP9/TPS1/BP, (**d**) mPP9/TPS1, (**g**) mPP8/TPS2, and (**j**) mPP8/TPS2/BP. Cross-sectional SEM images of (**b**) PP9/TPS1/BP, (**e**) mPP9/TPS1, (**h**) mPP8/TPS2, and (**k**) mPP8/TPS2/BP. Cross-sectional optical microscope images of (**c**) PP9/TPS1/BP, (**f**) mPP9/TPS1, (**i**) mPP8/TPS2, and (**l**) mPP8/TPS2/BP.

Figure 2a shows the side view and Figure 2b,c present the cross-sectional images of PP9/TPS1/BP produced via in situ mixing of unmodified PP and TPS. The resulting fibers exhibit irregular extrusion, fine pores, and rough cross-sections. Additionally, PP8/TPS2/BP, with a higher TPS content, was also unsuitable for spinning and was excluded from further analysis. These results indicate that higher TPS content exacerbates instability during spinning.

Also, BP was incorporated into the PP/TPS composites to enhance fiber biodegradability. However, BP acted as an impurity during blending, adversely affecting spinnability [50,67]. To mitigate this effect, strategies to improve miscibility between polymers and additive during melt-spinning were explored.

A masterbatch system was implemented to enhance compatibility, comprising the pre-mixing of a portion of the PP with TPS at a 1:1 ratio. This approach, widely used to disperse additives uniformly within a polymer matrix [68,69,70,71], improved compatibility between PP and TPS [19,66,72]. Furthermore, hydrophilic modification of PP (mPP) was performed by introducing hydrophilic functional groups into PP chains, enhancing adhesion and interaction with hydrophilic materials [73,74,75].

Figure 2d show the side view and Figure 2e,f illustrate the cross-sectional images of mPP9/TPS1 fibers, confirming that hydrophilic modification effectively improved PP-TPS miscibility. These fibers exhibited uniform thickness and smooth cross-sections. Although mPP8/TPS2, containing more TPS, displayed slightly reduced uniformity compared to lower TPS content in one, the modification enabled successful spinning with a little discontinuity (Figure 2g–i). The side view and cross-sectional images of mPP8/TPS2/BP in Figure 2j–l emphasize significant improvements in spinnability, even with 20% TPS content and BP addition. While the fiber displayed rough and irregular surface morphologies, it demonstrated melt processability with little internal voids.

The primary melt-spinning tests confirmed that the masterbatch system and hydrophilic modification significantly enhanced fiber processability. Notably, mPP9/TPS1 exhibited excellent spinnability without discontinuity when elongated over two. In addition, mPP8/TPS2/BP, incorporating higher biodegradable content, demonstrated successful melt processability due to the applied modifications.

Based on these results, the optimal melt-spinning conditions were achieved for the compositions of PP/TPS mass ratios of 9:1 (mPP9/TPS1) and 8:2 (mPP8/TPS2, mPP8/TPS2/BP). Stable fiber formation was confirmed under a spinning temperature of 200 °C, screw speed of 7–9 rpm, and a draw ratio of 2.35. The specific processing parameters and material compositions can be found in Section 2.3 and Table 1.

### 3.2. Thermal Properties of the Biodegradable Fibers

**Figure 3:**
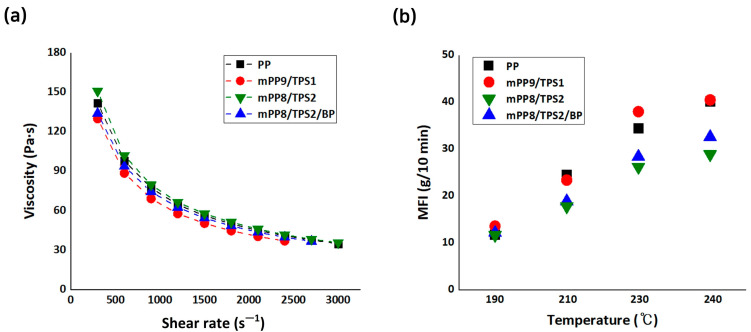
(**a**) Viscosity curves of polymer and polymer blend melts obtained from capillary rheometer; (**b**) melt flow index (MFI) curves of polymer and polymer blend melts.

The thermal properties of the blended materials were analyzed using a capillary rheometer and MFI to investigate the factors influencing the processability of the biodegradable fibers. The viscosity of virgin PP, mPP9/TPS1, mPP8/TPS2, and mPP8/TPS2/BP was measured under varying shear rates (Figure 3a). Virgin PP, known for its excellent spinnability, was selected as a reference material to establish melt-spinning parameters.

All four samples exhibited high viscosity at low shear rates, with viscosity decreasing as the shear rate increased; this behavior is characteristic of molten polymers, which act as non-newtonian fluids exhibiting shear-thinning. The shear-thinning occurs as polymer chain entanglements and molecular arrangements relax under shear stress [76]. The viscosity profiles of the studied blends resembled that of virgin PP, indicating that polymer-like behavior tends to stable spinnability under processing-induced stresses.

MFI, a more practical indicator for evaluating fiber melt-spinning performance, was measured for all samples at 190, 210, 230, and 240 °C (Figure 3b). The MFI of mPP9/TPS1 quite matched that of virgin PP, signifying its similar melt-spinning characteristics. This correlation between MFI and processability suggests that mPP9/TPS1 could serve as an industrially viable alternative to virgin PP for biodegradable fiber production.

In contrast, the other samples with higher TPS or BP content exhibited distinct MFI values compared to virgin PP, indicating slightly altered melt-spinning properties. However, combining these samples with mPP9/TPS1 could potentially yield fibers’ both biodegradability and enhanced melt-processability. The results emphasize the importance of processing techniques to optimize both spinnability and biodegradability in industrial fiber applications.

### 3.3. Sheath-Core Biodegradable Fiber Preparation

**Figure 4:**
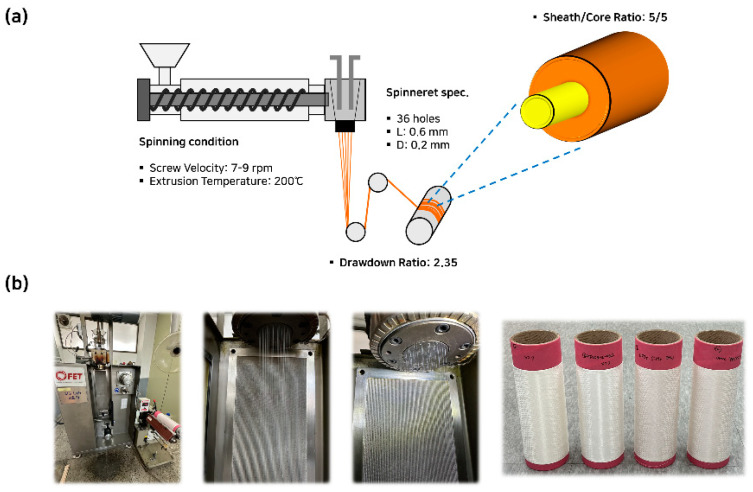
Sheath-core PP/TPS blend fiber preparation; (**a**) schematic illustration to exhibit the conditions of melt-spinning process; and (**b**) images of ongoing melt-spinning process and the fabricated sheath-core fibers.

To endow both spinnability and biodegradability in a fiber, a sheath-core configuration was employed, with the sheath and core materials in a 5:5 ratio (Figure 4a). mPP9/TPS1 was selected as the core material due to its excellent spinnability, while materials with higher biodegradable content, mPP8/TPS2 or mPP8/TPS2/BP, were chosen for the sheath to ultimately maximize biodegradability and process efficiency. Two types of sheath-core fibers were designated: [PP/TPS BFs: (s) mPP8/TPS2–(c) mPP9/TPS1] and [PP/TPS/BP BFs: (s) mPP8/TPS2/BP-(c) mPP9/TPS1], ensuring a balance between spinnability and biodegradability (Figure 4b).

**Figure 5:**
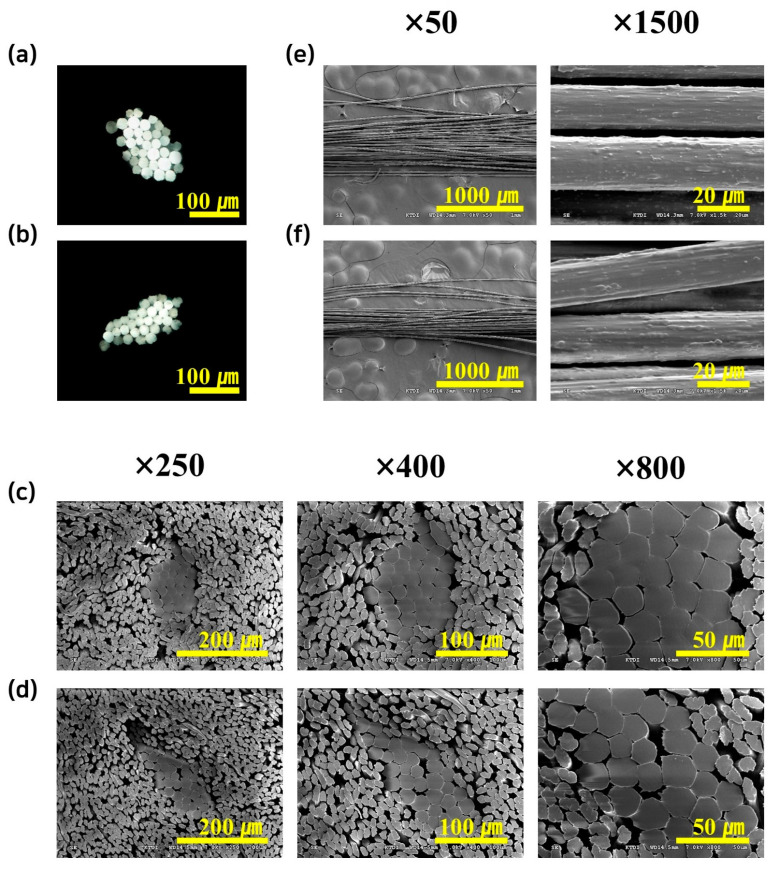
Optical microscope images (**a**,**b**) and SEM images (**c**–**f**) of sheath-core fibers. Cross-sectional optical microscope images of (**a**) PP/TPS BFs and (**b**) PP/TPS/BP BFs, cross-sectional SEM images of (**c**) PP/TPS BFs and (**d**) PP/TPS/BP BFs, and side view images of (**e**) PP/TPS BFs and (**f**) PP/TPS/BP BFs.

Cross-sectional and lateral views of both BFs were observed using optical microscopy and SEM to evaluate material compatibility after melt-spinning. The PP/TPS BFs displayed smooth cross-sections with no visible pores, indicating uniform fiber formation and strong internal durability (Figure 5a,c,e). Meanwhile, the PP/TPS/BP BFs showed slightly rough lateral surfaces but still maintained continuous extrusion with rare breaks, demonstrating reasonable spinnability (Figure 5b,d,f). Notably, no obvious separation was observed between the sheath and core, confirming structural stability and high level of material compatibility. Here, a custom and unique spinneret design was used to prevent interface formation between sheath and core when both comprised the same materials, enabling uniform integration. The spinneret design only offered high amounts of biodegradable contents in the BFs.

**Figure 6:**
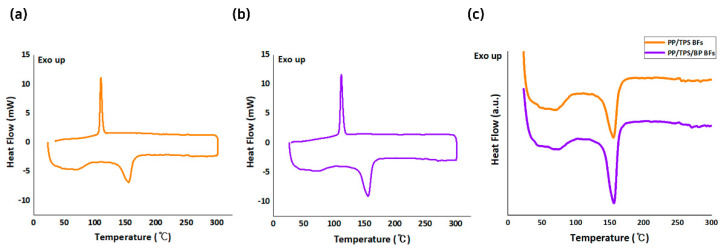
DSC curves of (**a**) PP/TPS BFs and (**b**) PP/TPS/BP BFs, and (**c**) heating curves of PP/TPS BFs and PP/TPS/BP BFs.

The melting points of the BFs were analyzed to further evaluate compatibility. DSC measurements revealed single melting peaks at 155.15 °C for PP/TPS BFs (Figure 6a) and 156.09 °C for PP/TPS/BP BFs (Figure 6b), respectively. For further comparison, heating curves of PP/TPS BFs and PP/TPS/BP BFs were presented (Figure 6c). The presence of a single melting temperature for each fiber type, despite their sheath-core structures, indicates no phase separation between the sheath and core. This result demonstrates a high level of compatibility between the materials as well as the thermal and structural stability of the fibers. These findings verify that the prepared BFs possess desirable mechanical and thermal properties, high biodegradability, and structural integrity, making them suitable for industrial applications.

### 3.4. Industrial Requirement Confirmation of the Biodegradable Fibers

**Figure 7:**
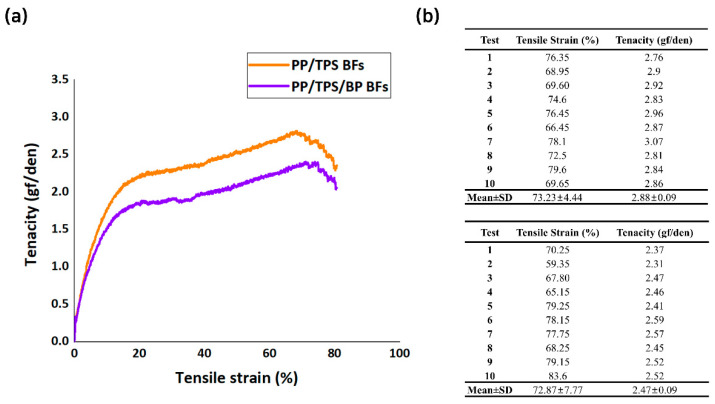
Tenacity–tensile strain curves of (**a**) PP/TPS BFs and PP/TPS/BP BFs. (**b**) The maximum tensile strain and tenacity values for each test of PP/TPS BFs (**top**) and PP/TPS/BP BFs (**bottom**).

The mechanical properties of the developed BFs, including tensile strength and elongation performance, were evaluated to assess their suitability for meeting conventional industrial requirements. Tensile strength tests offered that the PP/TPS BFs displayed a tenacity of 2.88 ± 0.09 gf/den and a tensile strain of 73.23 ± 4.44%, while the PP/TPS/BP BFs showed a tenacity of 2.47 ± 0.09 gf/den and a tensile strain of 72.87±7.77% (Figure 7a). The results, averaged over 10 trials, are noted in Figure 7b.

The tensile test results indicated that the PP/TPS BFs without BP achieved slightly better tenacity and tensile strain than the BP-containing fibers. However, the performance difference was quite small. This suggests that the sheath-core structure effectively compensates for the minor reduction in mechanical properties caused by the limited miscibility introduced by BP, while significantly enhancing other aspects of fiber performance. Moreover, despite the high TPS content in each sheath material, the fibers can still be spun and drawn successfully. This attributed to the excellent spinnability provided by the high PP content in the core, which ensures process stability and mechanical performance.

The mechanical properties of PP/TPS BFs and PP/TPS/BP BFs fabricated in this study, the fibers reported in previous studies, and tenacity and tensile strength values are presented in Table 2. PP/Polyvinyl Alcohol (PVA), PP/PLA, and PP/Cellulose are fibers fabricated by blending PP with representative biodegradable polymers, while PP refers to virgin PP, which is non-biodegradable. PP/TPS BFs and PP/TPS/BP BFs exhibit higher tenacity compared to PP/PVA and PP/PLA. Given that TPS generally has inferior mechanical properties compared to PVA and PLA, this result suggests that the optimized spinning conditions and the introduction of the sheath-core structure contributed to the improvement in tenacity of these BFs. Compared to PP/Cellulose, PP/TPS BFs and PP/TPS/BP BFs show slightly lower tenacity and tensile strain values. A similar trend is also observed in comparison with virgin PP, which can be attributed to the high PP content. Consequently, from this perspective, it can be emphasized that the BFs possess mechanical performance comparable to that of conventional PP-based fibers.

**Figure 8:**
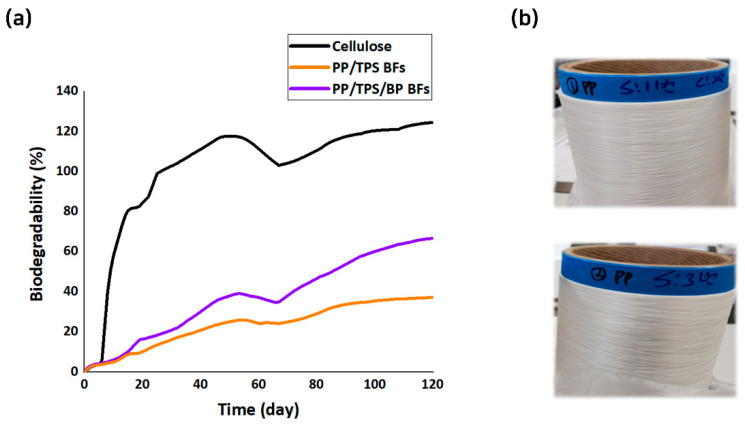
(**a**) Biodegradation curves of cellulose and sheath-core fibers. (**b**) Images of fabricated sheath-core fibers of PP/TPS BFs (**top**) and PP/TPS/BP BFs (**bottom**).

Lastly, the tradeoff effect between decreased spinnability and improved biodegradability with BP addition was assessed through a biodegradability test. This test measured the amount of carbon dioxide generated during the biodegradation of the fibers over time. Both types of prepared BFs (Figure 8a) were tested with cellulose, which was used as a positive reference material for biodegradation. After 115 days under standardized testing conditions, which is enough of a period for the biodegradability confirmation [82], the PP/TPS and PP/TPS/BP BFs achieved biodegradation rates of 37.00% and 65.93%, respectively (Figure 8b). Under the same conditions, the reference cellulose exhibited the highest biodegradation rate at 123.85%. These results are the experimental demonstration that blending petroleum-based polymers with bio-based components contributes to the development of biodegradable materials.

Notably, the PP/TPS/BP BFs displayed significantly higher biodegradability compared to the without-BP one. This is likely due to the inclusion of the BP during fiber production, which acts as a catalyst to accelerate the breakdown of the material. Under landfill composting conditions, the oxo-mPP in the BP-containing fibers undergoes aerobic biodegradation more readily, fragmenting into smaller pieces and thereby expediting the overall biodegradation process [38,39]. By contrast, the relatively lower biodegradation rate of PP/TPS BFs appears to be due to their reliance solely on TPS content, the bio-based component, without the additional accelerating effect of BP.

This study confirms that BP accelerates the degradation of PP/TPS BFs. As degradation progresses, polymers, including biodegradable polymers, lose mechanical strength. This process is influenced by factors such as moisture, UV radiation, weathering, temperature, oxygen, microbes, and pH [83,84,85]. Especially, BP promotes oxidative degradation, potentially accelerating mechanical deterioration. Previous studies showed that PP films with pro-degradant additives (PDAs) lost mechanical strength after thermo-oxidative aging (70 °C, 100 h) [86]. However, with a small amount of PDA (1.0%), high mechanical integrity was maintained throughout the experimental period. Similarly, PP/PLA blends exposed to 150 days of weathering showed reduced thermal stability and mechanical properties, but PP-g-MA, used as a compatibilizer, improved weathering resistance [87]. While this study did not conduct long-term mechanical performance testing, the BFs are expected to lose strength as biodegradation accelerates. However, prior research suggests that mPP and small amounts of BP may help maintain mechanical performance.

## 4. Conclusions

This study successfully developed biodegradable fibers with suitable mechanical properties though melt-spinning techniques, a sheath-core configuration, and advanced polymer modification strategies. The incorporation of hydrophilic mPP, combined with a masterbatch approach, effectively enhanced the compatibility between mPP and TPS, overcoming the inherent immiscibility challenges of the blend. The thermal and mechanical analyses confirmed that mPP9/TPS1 fiber exhibited melt-spinning processability and tensile properties comparable to virgin PP, triggering it as a viable alternative for industrial applications. Additionally, the sheath-core structure, with mPP9/TPS1 as the core and mPP8/TPS2 or mPP8/TPS2/BP as the sheath, achieved a balance between spinnability and biodegradability. The customized spinneret design enabled uniform fiber formation, as evidenced by single melting peaks in DSC measurements and SEM observations showing no phase separation between the sheath and core.

While BP addition slightly reduced spinnability, it significantly enhanced the biodegradation rate, with PP/TPS/BP fibers achieving 65.93% biodegradation after 115 days, compared to 37.00% for PP/TPS fibers. This emphasizes the effectiveness of blending petroleum-based polymers with bio-based components and biodegradation promoters in achieving environmentally sustainable fiber solutions. These studies demonstrate the potential of these biodegradable fibers to meet industrial requirements while addressing sustainability challenges.

## Figures and Tables

**Table 1:** Composition and mechanical properties of fibers.

		Sample Code				
		PP9/TPS1/BP	PP8/TPS2/BP	mPP8/TPS2/BP	mPP8/TPS2	mPP9/TPS1
Material Content (%)	M/B	-	-	40	40	20
	PP	90	80	-	-	-
	mPP	-	-	60	60	80
	TPS	10	20	20	20	10
	BP	2	2	2	0	0
Denier (D)		5.33	-	6.12	2.90	2.65
Tenacity (gf/den)		0.44	-	0.79	1.58	2.20
Tensile strain (%)		63.6	-	203.3	70.3	63.6
MI (230/2.16) (g)		38.8	44.4	39.3	29.3	34.1
Melting point (°C)		149.72	-	154.84	156.46	154.95

M/B consists of an mPP/TPS ratio of 1:1. The mPP content was calculated as the final mPP content minus the mPP included in the M/B. The TPS content was determined based on the TPS included in the M/B. TPS contains both a plasticizer and an immobilizer.

**Table 2:** Comparison of the mechanical properties of fibers.

Fibers	Tenacity (gf/den)	Tensile Strain (%)	Reference
PP/TPS BFs	2.88 ± 0.09	73.23 ± 4.44	-
PP/TPS/BP BFs	2.47 ± 0.09	72.8 ± 7.77	-
PP/PVA (70/30)	1.39 ± 0.19	-	[77]
PP/PLA (50/50)	1.47 ± 0.09	123.82 ± 9.69	[78]
PP/Cellulose (1.25)	3.18 ± 0.08	about 90	[79]
PP	3.5–5.5	40–100	[80]
PP	3.91	-	[81]

## Data Availability

The original contributions presented in this study are included in the article. Further inquiries can be directed to the corresponding authors.
